# Child-Centred Care in HIV Service Provision for Children in Resource Constrained Settings: A Narrative Review of Literature

**DOI:** 10.1155/2019/5139486

**Published:** 2019-11-26

**Authors:** Chipo Mutambo, Kemist Shumba, Khumbulani W. Hlongwana

**Affiliations:** ^1^The Discipline of Public Health Medicine, School of Nursing and Public Health, University of KwaZulu-Natal, Durban, South Africa; ^2^The Discipline of Psychology, School of Applied Human Sciences, University of KwaZulu-Natal, Durban, South Africa

## Abstract

**Introduction:**

Child-centred care approaches are increasingly gaining traction in healthcare; and are being applied in the delivery of HIV care for children in resource constrained settings. However, very little is known about their potential benefits.

**Methods:**

We synthesised literature from primary and secondary publications exploring the philosophical underpinnings of the concept of child-centred care, and its application to HIV service delivery for children in resource constrained settings. We concluded the review by suggesting a conceptual framework for mainstreaming and integrating child-centred care approaches in the management of HIV in resource constrained settings.

**Results:**

The philosophical underpinnings of child-centred care stem from human rights (child-rights), holism, the ecological model, and life-cycle approaches. Although there is no standard definition of child-centred care in the context of HIV, the literature review highlighted several phrases used to describe the “child-centredness” of HIV care for children. These phrases include: (i) Respect for child-healthcare rights. (ii) Using the lifecycle approach to accommodate children of different ages. (iii) Provision of age-appropriate HIV services. (iv) Meaningful participation and inclusion of the child in the healthcare consultation process. (v) Using age-appropriate language to increase the child's understanding during healthcare consultations. (vi) Age-appropriate disclosure. (vii) Primary caregiver (PCG) participation and preparation (equipping the PCGs with information on how to support their children). (viii) Creation of a child-friendly healthcare environment. (ix) Consideration of the child ecological systems to have a holistic understanding of the child. (x) Partnership and collaborative approach between children, PCGs, and healthcare workers (HCWs).

**Conclusion:**

Child-centred care approaches can potentially increase child-participation, promote positive health outcomes and resilience in children living with a communicable, highly stigmatised and chronic condition such as HIV. More evidence from controlled studies is required to provide concrete results to support the application of child-centred care approaches in HIV care services.

## 1. Background

Historically, a child did not have any rights in healthcare, due to the traditional paternalistic healthcare worker (HCW) driven and disease-focused approach to care [1, 2]. Stakeholders, including HCWs and primary caregivers (PCGs) believed that children were incapable of either coherently communicating their health issues and needs during healthcare consultations or contributing in any manner in the decision-making processes regarding their care [2, 3]. The United Nations Convention on the Rights of the Child (UNCRC) (1989) [4] challenged this belief by affirming children's right to participate in healthcare, thereby changing the status quo [1]. The UNCRC gave substance to the rights of children and affirmed that children under the age of 18 years should be afforded age-sensitive healthcare [1, 2]. This watershed convention gave children a voice in healthcare service. This unconventional approach by the UNCRC constituted a paradigm shift from the pervasive paternalistic behaviour demonstrated by doctors and endorsed by the PCGs of children [1, 2].

According to Article 12 of the UNCRC, children are entitled to being involved in their healthcare. However, this involvement should be in accordance with their evolving capacities, cognisance of the scale of decisions to be made and competencies thereof [1]. Article 12 emphasises the importance of a “broad-based approach” to children's healthcare, which should consider the child's cognitive development and maturity, as well as their developmental stage [1]. Generally, the UNCRC sets out four broad principles to ensure the protection of children's rights to healthcare. These principles are; nondiscrimination, best interests of the child, the right to life, survival and development, and the right of the child to express his/her views [4, 5]. The UNCRC's clarion call to promote child-rights provides a blueprint for the emergent healthcare philosophy of “child-centred care,” or “child-friendly care” [5]. Although the concept of child-centred care is growing in prominence, it still lacks a concrete definition, application, and conceptual clarity [3, 6, 7]. This is due to the diverse interpretations offered in different contexts. There is still confusion regarding what child-centred care entails. The confusion emanates from the overlap between child-centred care and other pervasive healthcare approaches, including patient-centred care and family-centred care [3, 6, 7].

There are arguments suggesting that the concept of child-centred care is already enshrined in the definition of patient-centred care [3, 6]. According to the Institute of Medicine (IOM), patient-centred care is the provision of, “*care that is respectful of, and responsive to, individual patient preferences, needs and values, and ensuring that patient values guide all clinical decisions*” [8]. Some scholars add that the concept of patient-centred care is embedded in the holism paradigm, which views patients as biopsychosocial and physiological beings [9, 10]. When applied to healthcare service delivery, holism suggests that services should be responsive and respectful to the needs of individual patients, and must be tailored to address the biological, psychological, and social dimensions of disease [9–11]. With regard to clinical decision-making, holism suggests that individual patients should be given the autonomy to choose and voice personal preferences, and that the partnership approach to engage in a decision-making process that is inclusive of the individual patient, the patients' family and the HCW must be applied [9, 10]. The idea of holism within patient-centred care enshrines the “nothing about me, without me” mantra [9, 12].

Some studies synonymise child-centred care with family-centred care [3, 6]. The family-centred care philosophy posits that the family is the unit of care and that healthcare for children is a joint effort between PCGs, other family members, and the HCWs who are given the responsibility of ensuring that the child is provided with care [5]. Although both concepts are cut from the same cloth of “centredness”, family-centred care is widely celebrated and ingrained in various healthcare policies and guidelines [6], yet child-centred care is not, and remains largely elusive. However, recent publications have criticised family-centred care approaches for perpetuating HCW paternalistic ideologies and PCG dominance, thus creating an asymmetrical relationship between the child, HCW and PCG, which stifles children's right to participation and decision-making in accessing healthcare services [6, 7, 9]. Additionally, family-centred approaches still perceive children as being minors that are in need of protection from PCGs, characterised by cognitive immaturity and unable to contribute to decision-making regarding their care [5, 8]. Inadvertently, this forces children to become passive recipients of healthcare services. Some scholars have blamed the ambiguity of the concept of family-centred care for creating role conflict between HCWs and PCGs [7]. Despite the widespread reference to family-centred care in policy documentation and guidelines, there is still no concrete evidence of its effectiveness [6, 9].

On the other hand, the concept of child-centred care can be viewed as a customisation of the concept of patient-centred care that seeks to cater for the needs of children. It addresses some of the complexities related to children that may have been overlooked by patient-centred care [6, 12, 13]. This distinction provides an allowance for the inclusion of child rights affirmed in the UNCRC's child-centred care philosophy [7, 14]. Like patient-centred care, key principles underpinning the concept of holism are also applicable to child-centred care. However, there are additional considerations introduced by the sheer nature of what it means to be a child, and childhood as a human developmental stage [13]. Child-centred care celebrates childhood and acknowledges the cognitive, legal, and cultural challenges and limitations associated with childhood. Child-centred approaches address these gaps by firstly recognising children as “agentic beings” with the ability to actively take part and influence their healthcare and make decisions that affect their care [3]. Child-centred care is not only rooted in holism but it also borrows some constructs from the rights-based philosophy [1, 14]. Within the ambit of the rights approach;

- Childhood is no longer viewed as a homogenous state and differs cross-culturally [13],
- Care provided to children is differentiated according to their age, gender, ethnicity, developmental stage, and maturity [13, 15–17],
- Children are seen as agentic beings with the capability of being social actors with the ability to influence the world around them [7, 13], and
- Children have rights, opinions, and unique experiences that grant them the right to participate in decision-making that affects them [5, 13, 18].

The pro-child-rights approach addresses both the passive nature of family-centred care in healthcare matters that involve children, and the generalist nature of patient-centred care because it is responsive to the specific needs of children. It affords children the opportunity to articulate their needs and sensibilities. Child-centred care transforms children from being bystanders to active players by increasing their autonomy and self-determination; it strengthens their resilience [1]. This approach also ensures that the voices of children are not stifled and barred from entering the dominant discursive spaces. It celebrates their childhood in a manner that assures their best interests [1, 17, 18]. Child-centred care acknowledges the legal status of children as minors under the care of PCGs, but gives these adults the leeway to perform the role of the child's advocate, intermediary, and interpreter during healthcare consultations [1].

The application of child-centred care to the delivery of HIV services is still very limited. However, there is evidence of the application of some of its constructs to HIV programmes for children [19–21]. Furthermore, the concept has still not been defined extensively especially in the context of HIV, although some scholars are gradually realising the value of tailoring HIV services to be in sync with the needs of the child [6, 7, 17, 21, 22]. A good example of such an intervention is the promotion of status disclosure for children living with HIV conducted in Namibia where a cartoon-based storybook was used to facilitate disclosure [23, 24]. By applying the right to both information and participation, healthcare providers are providing children with age-appropriate information to facilitate disclosure and potentially improve medication adherence and achieve positive health outcomes [25–27]. The sharing of information through consultative sessions between HIV seropositive children, HCWs, and PCGs has been shown to forge partnerships between the parties involved, and promote transparency and truthfulness, which is key to promoting status disclosure [25–27]. In addition to improving medication adherence, disclosing children's HIV seropositive status to these minors as early as possible is vital to preventing the inadvertent transmission of the virus when they eventually become sexually active [25, 26, 28].

While there is a great deal of evidence on the application of family-centred care and patient-centred care to the management of HIV in children, studies on the application of child-centred care in that same context are still very limited. Our search for narrative reviews, scoping reviews, or systematic reviews on MEDLINE, PubMed, and Google Scholar databases, prior to conducting this study, did not yield any results, hence we considered it necessary to synthesise the literature from primary and secondary publications, describing the philosophical underpinnings of the concept of child-centred care, and its application to HIV service delivery for children. This study is both timely and apt, in light of the global HIV agenda of differentiated care models for children living with HIV [29]. Furthermore, it adds to the limited body of evidence on the application of alternative care approaches for children living with HIV.

## 2. Methods

We retrieved relevant primary, secondary, and tertiary literature from electronic databases including; MEDLINE, PubMed, and Google Scholar. We also reviewed the reference lists of retrieved articles to identify additional relevant articles. To increase the sensitivity of the search words, we used keywords and MeSH (Medical Subject Headings) terms and reviewed the reference lists of studies identified. We synthesised literature from sources that showed evidence of the application of the child-centred approach in the management of HIV among children and clearly defined elements of the concept of child-centred care. Key search words used during the search included, but were not limited to the following: *Child-centred care, OR Child-centred care, OR Child-engagement or Child participation OR Child-engagement OR Patient-Cantered for Children OR Person-centred Care for children AND HIV OR HIV Care OR HIV psychosocial support AND Poor Resource Countries OR Low resource Settings OR Sub-Saharan Africa.*

In the context of this study, HIV services for children included; HIV counselling and testing (HCT), anti-retroviral therapy (ART) initiation, treatment adherence, disclosure, counselling and support. The term “children” in this study refers to young people between the ages of 0–17 years, in accordance with the UNCRC (1989) [4].

## 3. Ethical Considerations

This study used existing published literature; therefore, no ethical approval was sought. However, this literature review was part of a larger doctoral study approved by the University of KwaZulu-Natal's Biomedical Research Ethics Committee (BREC) (Ref. No. BE298/18) and the KwaZulu-Natal Department of Health (Ref. No. KZ_201809_011).

## 4. Results and Discussion

The literature review yielded five themes related to the application if child-centred care to HIV service for children. These are;

(a) Child-centred communication with children living with HIV.
(b) Communicating with PCGs of HIV seropositive children.
(c) Applying play therapy as a child-centred technique for enhancing communication with HIV seropositive children.
(d) Modifying the healthcare environment for the benefit of children.
(e) Capacity building among HCWs delivering HIV services to children.

**Figure 1:**
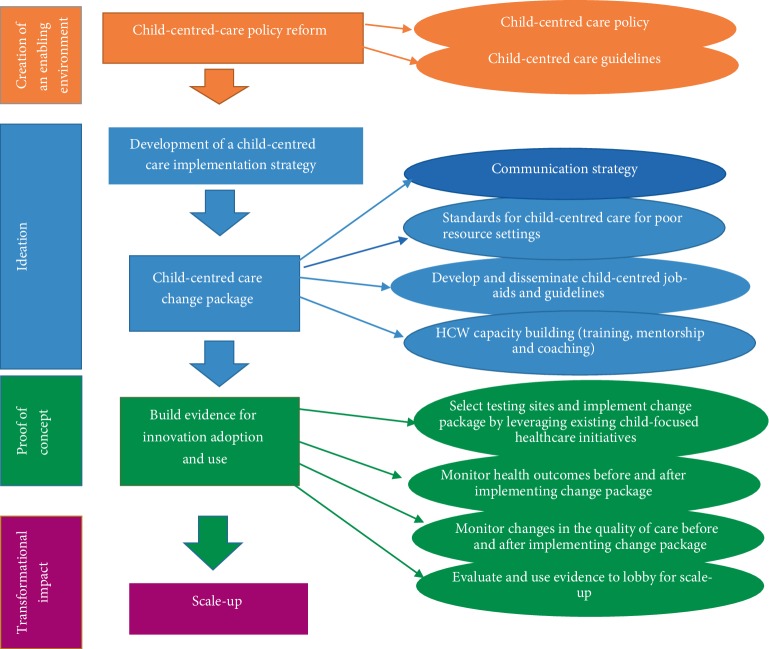
Proposed child-centred care implementation framework for resource constrained settings.

An analysis of these themes yielded a compilation of phrases used to describe the “child-centredness” of HIV care for children. In addition, several barriers to the application of child-centred care to HIV seropositive children in resource constrained settings are also themed and briefly discussed. In closing, the authors suggest a “child-centred care, implementation framework” for mainstreaming and integrating child-centred care approaches into HIV programmes implemented in resource constrained settings (Figure 1).

### 4.1. Applying the Concept of Child-Centred Care to HIV Service Provision

#### 4.1.1. Child-Centred Communication with HIV Seropositive Children

Generally, HCWs have a moral and ethical obligation to provide children with comprehensive healthcare services, and ensure their involvement and participation in their health care journey [30, 31]. It is important to note that the main actor in the child-centred care process is the child. Therefore, more time and effort should be spent communicating with the child to ensure that they understand their condition and strive to manage it well. The child-centred care process also ensures that HCWs and the child's PCG will support the child to manage the illness. Thus the communication process must encourage child-participation in the consultation process, affording them the opportunity to freely communicate, and air their concerns, ask questions, and be answered using language that they understand [26]. This sharing of information and consultative interaction between children, their HCWs, and PCGs have been shown to forge partnerships between the parties involved and promote transparency, and truthfulness, thus eliminating competition and conflict [26]. In addition, the child-centred care approach encourages HCWs to spend considerable time with children during consultations. As the HCW takes time to discuss with both the PCG and the child about an agreeable and appropriate care plan, doing so reassures the child and the PCG that the HCW is both attentive and genuinely concerned with the welfare of the child, while being respectful of the PCG's role [1]. In this way, child-centred healthcare is not only empowering but also increases children's participation and asserts their rights to be heard.

There is a plethora of evidence suggesting that effective communication with HIV seropositive children can marginally improve their treatment adherence, reduce adverse events, and reduce stress and anxiety [1, 16, 18, 26, 32]. Moreover, communicating health messages to HIV seropositive children has proved to be effective in improving their psychological and behavioural outcomes and in some instances, it has been proved to promote self-care [33]. In addition, effective communication between HCWs, PCGs, and children creates an enabling environment for early HIV seropositive status disclosure to children to prevent inadvertent transmission of the virus later in life when they become sexually active and it also improves medication adherence.

Despite rich evidence on the benefits of child-centred HCW communication with HIV seropositive children in healthcare settings, HCWs still face communication challenges [5, 19, 34]. Several studies in sub-Saharan Africa have reported that HCWs lack effective communication skills to enable them to provide HIV counselling to children [35]. The child developmental continuum was a key barrier to effective communication, since HCWs struggled with adjusting messages for different age groups, owing to the lack of requisite communication skills for different developmental stages [5]. Levetown et al. [30] propose communication that is nonthreatening, to reduce anxiety and intimidation among children, suggesting that these are practical behaviour and communication skills.

Levetown et al. [30] suggest that a discussion with the child should ensue and a broad topic on a nonthreatening subject, using a language that is understandable to the child must be initiated. Further, it is critical to pay attention to nonverbal cues, tone, and actively listen to children [18, 36]. In that respect, communication aids such as games, drawings, and stories to make the communication process more interesting and understandable for the child are proposed. These help to mitigate the effects of children's short concentration span [30]. A good example is the HIV *Disclosure Storybook* developed in Namibia, where cartoons are used as a job aid for providing disclosure health education to children. This book conveys information about HIV to children in a developmentally appropriate manner, using child-centred language and metaphors such as; “body soldiers”, referring to CD4 cells, “medicine” referring to ARVs and “bad guys”, which refers to the human immune-virus [37].

#### 4.1.2. Communicating with the PCGs of HIV Seropositive Children

Several studies have shown that children need the support, and participation of PCGs in their healthcare experience [18, 19, 33, 38]. HCWs are therefore obligated to furnish PCGs with the relevant information regarding their child's illness, so that they can provide the necessary support to their child [5]. Research has shown that HCWs often face uncertainty when it comes to informing children about a health condition or medical procedure and opt to solely discuss the child's illness with the PCG [5]. Conversely, some studies have reported that PCGs prefer that HCWs only communicate to them regarding their children's illness as they feel that they should protect their children from information that can potentially cause them emotional harm [5]. Studies in South Africa have found that PCGs of HIV positive children do not disclose to their children to protect them from psychological trauma, worry and depression, stigmatisation, gossip, and diminished will to live [19, 39, 40]. While some PCGs are wary of disclosing to their children, others are willing, but lack capacity to do so, thereby making it difficult for them to explain and answer questions which the child might ask [19, 40].

#### 4.1.3. Applying Play Therapy as a Child-Centred Technique for Enhancing Communication with HIV Seropositive Children

One of the rights enshrined in the UNCRC is the right to rest and play [13, 41]. Recognising that play is the universal language for children has led to its application in psychosocial interventions to reduce medical trauma among children. This approach is referred to as the play therapy [42]. Play therapy in the context of healthcare is defined as a therapeutic technique used in healthcare settings to communicate with children receiving health services to reduce trauma, and increase the child's understanding of procedures and their illness [43]. The technique considers the child's age, cognitive development, and health condition [41, 43]. It also allows children to freely express themselves during healthcare consultations [41]. Play therapy has been used successfully to prepare children for surgery or other unpleasant medical procedures [41, 43].

While the efficacy of play therapy in supporting children through traumatic events remains a subject for further research, there is a growing body of evidence indicating that it can have a positive effect on children who have experienced trauma, particularly if their parent or PCGs are involved [44]. Several studies have established that play-based techniques help children to become more involved in the therapeutic process, enhance the relationship between the child and the HCW, and create an overall positive experience [41, 42, 45–47]. In studies conducted in Nigeria [48] and South Africa [49], mothers and PCGs specifically identified play areas as important features when designing a clinic setting. Evidence points at play therapy being a helpful tool for children to express their emotions and articulate their concerns in a healthy way, build self-esteem, and develop coping mechanisms [50].

#### 4.1.4. Modifying the Healthcare Environment for the Benefit of Children

Empirical evidence supports the use of visual art and play areas inside a healthcare setting to marginally improve both the children's healthcare experiences and overall health outcomes [16, 19, 51], in line with the child-friendly space (CFS) concept recently introduced by humanitarian child protection agencies [52]. The Child Protection Working Group describes a CFS as one that “supports the resilience and well-being of children and young people who have experienced disasters through community organized, structured activities conducted in a safe, child-friendly, and stimulating environment” [53]. The concept of child-friendly spaces (also referred to as “safe spaces”, “child-centred spaces” and “child protection centres”) is centred on the children's need for a protected environment, in order to learn, express themselves, build self-esteem, socialise, and play—all of which are critical components of healthy psychosocial development [54]. These spaces also play a role in easing difficult transitions or providing a stable environment for children during difficult or traumatic experiences [55]. Child-friendly spaces also provide a contact point where HCWs and other professionals can assist children who are facing a threat. The concept encompasses not only the physical space but also the associated programmes that are delivered to children and their PCGs [56].

Initially developed to support children during times of humanitarian crisis, many international organisations, including UNICEF and Save the Children, have adopted CFS, as a key intervention for protecting children at risk [53, 57]. The CFS model is inherently adaptable to a variety of contexts and can be modified for different settings and age groups. However, there is currently a lack of data that rigorously evaluates the efficacy of child-friendly spaces. The existing data mainly focus on the impact of child-friendly spaces during humanitarian crisis situations, such as emergency and disaster [54]. An evaluation of ‘child-centred spaces' in Northern Uganda found that these spaces had a tangible benefit for children, translating into children who experienced less emotional distress, displaying fewer behavioural issues and better social skills. The ‘child-centred spaces' programme also resulted in children having improved knowledge about hygiene, communication skills, literacy, and numeracy [58]. The CFS also served as an information hub where children acquired knowledge that could then be disseminated to their community and peers. Other studies reported that access to a CFS helps decrease the sexual exploitation and abuse of children [59], relieves anxiety and withdrawal, and improves interactions between children and parents or PCGs [60].

Broad guidelines have been developed to guide the development of child-friendly spaces [55]. These guidelines suggest that a CFS should uphold the provisions of the United Nations Convention on the Rights of the Child treaty. CFS guidelines entail physical safety, participatory operated, culturally appropriate, and community inclusive, nondiscriminatory, diverse activities, and a commitment to employ sensitive and well-trained staff [57]. Ager and Metzler [61] add that successful evaluation of the impact of CFSs requires that a baseline study is conducted before implementing the CFS and subsequently monitor all the activities taking place before evaluation to compare the baseline with the outcomes observed over time [55]. These scholars also add that long-term follow-up is critical to creating the evidence-based benefits of establishing CFSs in a variety of settings to assist children at risk, but this must be inclusive and involve community inputs [61].

Other studies have noted that to be successful, a CFS must be child-centric and should not regard the child as a passive recipient of services, but rather an active partner in the design and delivery of child-friendly services [21, 61, 62]. Diverse and appropriate activities that focus on play, both as structured activities and free play, are essential to child health and psychosocial development [55]. This has been demonstrated in CFSs in both emergency and nonemergency settings [62]. In a comparison of case studies where CFSs were established in humanitarian emergencies in areas such as India and Sierra Leone, it was emphasised that CFSs needed to incorporate play therapy and creative activities to enhance effectiveness [63]. Song, dance, drawing, and drama are all suggested to enhance different skills, such as problem-solving, communication, and cooperation [55].

The value of using a CFS to assist children who are vulnerable to health-related crises including the HIV epidemic is gradually gaining traction in resource constrained settings [21, 64]. Children struggle with issues such as understanding what HIV is, and how it will affect them [65], disclosure of their own or a caregiver's HIV seropositive status [66], adherence to lifelong antiretroviral therapy (ART) regimens and a lack of entry points into the health care system for HIV testing, often perceiving adult HIV clinics as frightening and unwelcoming [67]. A lesson from an adolescent-friendly HIV clinic design in Cape Town is that many healthcare providers are viewed as having negative attitudes and are not trusted to maintain confidentiality—a potential training issue that can be addressed to help boost uptake of paediatric HIV services [68].

#### 4.1.5. Capacity Building of HCWs Delivering HIV Services to Children

To meet the constantly changing demands in managing HIV, HCWs need to be capacitated with the know-how, skills, and confidence to deliver child-centred services. Some studies have suggested that HCWs have very little or no understanding of existing guidelines for providing child-centred HIV testing services (HTS) and disclosure counselling [23, 69, 70]. A Ghanaian study found that HCWs were unsure of the language or approach to use particularly when providing counselling and health education during HTS, and whether to provide these to the child or to just have a discussion with the PCG only [35]. To mitigate these challenges, literature suggests that in-service training and mentorship of HCWs are necessary capacity building processes that can improve performance, quality of HIV healthcare services, and patient outcomes [71, 72]. In-service training is reportedly one of the strategic activities with ample financial support from funders supporting the fight against the scourge of HIV and AIDS [72].

### 4.2. Barriers to Child-Centred Care for HIV Seropositive Children

HCWs lack knowledge and understanding of child-centred approaches and how to apply them during health service delivery to HIV seropositive children [1]. Several studies report that HCWs lack knowledge about child-centred care, which affects their ability to effectively communicate to children of different ages [19, 25, 69, 73]. Consequently, this contributes to HCWs seeking to avoid engaging with children. When this happens, children are marginalised from their care, which results in the infringement of their right to participation, and a culmination of negative experiences of care, anxiety and poor understanding of their condition. Other related consequences include poor adherence to medical advice, medication adherence, and ultimately negative health outcomes. In addition, child-centred approaches require a *supportive healthcare environment, *which most healthcare spaces are unable to provide [21]. Other studies have suggested play areas for children in health institutions as a transformative measure towards child-centredness [19, 21]. *Lack of official guidelines* on how to provide child-centred care, is among the barriers to this HIV management approach [17, 19]. For child-centred care to become a reality in HIV care for children, there is a need for official guidance to standardise the care for all children attending healthcare facilities.

*HCW paternalism* presents as a barrier to child-centred care for HIV seropositive children [74]. This is because of the additional cognitive limitations related to understanding, reasoning and retention of information experienced by children, which compels HCWs to take a paternalistic stance when providing care to these children [74]. Studies suggest that HCWs have an ethical, legal and clinical obligation to support children's involvement in consultations and decision-making processes [25, 74]. Thus, paternalism acts against these ethical and clinical obligations and violate children's healthcare right to age appropriate-approaches, and developmental stage-appropriate information. It is also a breach of their right to participate and contribute to own care. To mitigate the negative effects of paternalism, a complete overhaul of the HCW's mindset is required. Training, mentorship and guidelines on child-centred care, life-cycle approaches and rights-based approaches have been suggested as measures for promoting the consideration of both patients' and HCWs' perspectives of healthcare delivery [25].

Defining the role of the PCG in child-centred care for children is not only necessary, but can be the biggest game changer in the care for children living with HIV. This is so because it can potentially address the barriers to disclosure, arguably one of the factors impeding medication adherence among children [69, 75]. Studies suggest that children desperately want to be involved in their care, but their PCGs make it difficult for them to do so [1, 19, 22]. Thus children need the support of their PCGs as they want them to take on the role of being their advocates, intermediaries, and interpreters during the healthcare consultation process [1, 20, 25, 34]. Moreover, children have highlighted that they prefer to have their PCGs present during the consultation process so that they can explain information which they might not have understood due to the HCW's communication style [1, 22].

### 4.3. Defining Child Centred Approaches in the Context of HIV

Although there is no standard definition of child-centred care in the context of HIV, the literature review highlighted several phrases used to describe the “child-centredness” of HIV care for children. These phrases include:

- Respect for child-healthcare rights [17, 21, 25].
- Using the lifecycle approach to accommodate children of different ages [17, 25].
- Provision of age-appropriate HIV services [25, 75].
- Meaningful participation and inclusion of the child in the healthcare consultation [25, 75].
- Using age-appropriate language to increase the child's understanding during healthcare consultations [13, 75].
- Age-friendly packaging of HIV messages to increase the child's understanding of own illness [13, 23, 25].
- Age-appropriate disclosure [23, 25].
- PCG participation and preparation (equipping the PCGs with information on how to support their child) [23, 25].
- Creation of a child-friendly healthcare environment [15, 25]
- Consideration of the child's ecological systems in order to have a holistic understanding of the child [17, 25].
- Partnership and collaborative approach between HCWs, PCGs and children [76]

### 4.4. Child-Centred Care, Implementation Framework in Resource Constrained Settings

Literature on the viable implementation of frameworks for scaling up health interventions is growing [77–81]. These frameworks focus on providing advice to policy-makers and funding agencies from low and middle income countries on how to scale-up public health interventions [82]. Similarly, we propose an implementation framework for mainstreaming and integrating child-centred care, in HIV service provision in resource constrained settings (Figure 1).

#### 4.4.1. Creating an Enabling Environment Through the Creation of Child-Centred Care Policy and Formal Guidelines

To create an enabling environment for the adoption of child-centred care, there is need for policy reform led by healthcare decision makers. Country specific healthcare policy development and guidelines are necessary to define, contextualise, and endorse child-centred care approaches as the standard of care for all children, including those living with HIV. Policy makers in resource constrained settings are scrambling to meet the Joint United Nations Programme on HIV and AIDS' (UNAIDS) 90-90-90 goals and are receptive to innovative quality of care interventions with a potential to positively contribute to the attainment of these targets. Therefore, innovative public health practitioners may use this information to lobby for policy reform and the development of guidelines for child-centred care.

#### 4.4.2. Development of a Child-Centred Care Implementation Strategy

Highlighting how the child-centred approach would be integrated and mainstreamed into existing HIV interventions and programmes would be developed next. It would also describe the key players and their roles (i.e. children living with HIV, HCWs, PCGs, families, healthcare institutions) in the application of the concept and a communication package.

#### 4.4.3. Child-Centred Carechange Package

The package spells out all the change ideas that need to be implemented to effectively introduce the concept of child-centred healthcare into the healthcare institution.

- *Deciding on standards for child-centred care in resource constrained settings:* To successfully lobby for the cultivation of a culture of child-centredness in HIV programmes on the frontline, there is need to decide on a standard definition of the concept and develop standards for measuring the “child-centredness” of healthcare institutions. It is this definition that should inform the development of standards for child-centred HIV care, which will contribute to establishing consistency or uniformity in all healthcare institutions providing HIV care to children.
- *Developing a communication strategy:* There is need to build awareness of the new ideas and provide technical support to those ready to adopt the ideas.
- *HCW capacity building:* Capacity building is important to increase knowledge, change attitudes, improve skills, and increase confidence to provide child-centred care, during HIV service delivery.
- *Development and dissemination of child-centred job-aids:* In addition, child-centred/child-friendly job-aids need to be developed and disseminated. Apparently, there is a need to create child-centred healthcare environments in existing healthcare facilities [17, 19].

### 4.5. Build Evidence for Innovation Adoption and Scale-Up

The final stage is testing of the change package in small pilot studies to build evidence for innovation adoption. To test the effect of the child-centred change package in healthcare facilities, there is a need to leverage existing child-focused healthcare initiatives that require such an approach for them to be successful. These include; Provider Initiated Counselling and Testing (PICT) programmes, HIV treatment programmes and immunisation and Integrated Management of Childhood Illnesses, which are primarily programmes for children. Leveraging the above-mentioned interventions present as low-hanging fruit for integrating and testing the viability of the child-centred approach. Data on HIV outcomes, patient satisfaction, patient health experiences, and quality of care must be collected before and after the intervention, and these data will be used to appraise the effect of the package. Evaluating the impact of this child-centred care package will generate data to convince public health leaders to endorse the approach and fund its scale-up.

## 5. Implications of the Study

This study reflects on the way child-centred approaches can potentially be applied in the care of children living with HIV, in order to improve the quality of care and mitigate challenges. From a quality of care perspective, child-centred care addresses key programmatic challenges that are not addressed by other care approaches, such as child-participation and child rights. It provides a life-cycle orientated approach, which acknowledges the needs of children at their various stages of development. This study also provides policy makers and public health decision-makers with pointers on how to mainstream and integrate child-centred care, approaches in the management of HIV.

## 6. Limitations of the Study

Due to the limited studies on child-centred care, our findings especially the proposed framework (Figure 1) arising from this study may not be convincingly appreciated by some policymakers and programme implementers. However, this study adds on to the limited body of knowledge and contributes to the growing debate on the value of child-participation in healthcare.

## 7. Conclusions

Child-centred care approaches are important in healthcare provision as they potentially increase children's participation, improve health outcomes, and promote resilience among children living with HIV, which is a communicable, highly stigmatised, and chronic condition. However, there is still a great deal of work to be done particularly with regard to comprehensively defining the concept, exposing its various facets and how it relates to patient-centred care and quality of care. Furthermore, additional evidence from controlled studies is required to provide concrete results that support the approach.
